# Multi-Targeted Mechanisms Underlying the Endothelial Protective Effects of the Diabetic-Safe Sweetener Erythritol

**DOI:** 10.1371/journal.pone.0065741

**Published:** 2013-06-05

**Authors:** Daniëlle M. P. H. J. Boesten, Alvin Berger, Peter de Cock, Hua Dong, Bruce D. Hammock, Gertjan J. M. den Hartog, Aalt Bast

**Affiliations:** 1 Department of Toxicology, Maastricht University, Maastricht, The Netherlands; 2 Global Food Research, Cargill, Wayzata, Minnesota, United States of America; 3 Cargill RandD Center Europe, Vilvoorde, Belgium; 4 Department of Entomology and UCD Comprehensive Cancer Center, University of California Davis, Davis, California, United States of America; University of Kansas Medical Center, United States of America

## Abstract

Diabetes is characterized by hyperglycemia and development of vascular pathology. Endothelial cell dysfunction is a starting point for pathogenesis of vascular complications in diabetes. We previously showed the polyol erythritol to be a hydroxyl radical scavenger preventing endothelial cell dysfunction onset in diabetic rats. To unravel mechanisms, other than scavenging of radicals, by which erythritol mediates this protective effect, we evaluated effects of erythritol in endothelial cells exposed to normal (7 mM) and high glucose (30 mM) or diabetic stressors (e.g. SIN-1) using targeted and transcriptomic approaches. This study demonstrates that erythritol (i.e. under non-diabetic conditions) has minimal effects on endothelial cells. However, under hyperglycemic conditions erythritol protected endothelial cells against cell death induced by diabetic stressors (i.e. high glucose and peroxynitrite). Also a number of harmful effects caused by high glucose, e.g. increased nitric oxide release, are reversed. Additionally, total transcriptome analysis indicated that biological processes which are differentially regulated due to high glucose are corrected by erythritol. We conclude that erythritol protects endothelial cells during high glucose conditions via effects on multiple targets. Overall, these data indicate a therapeutically important endothelial protective effect of erythritol under hyperglycemic conditions.

## Introduction

Chronic hyperglycemia in diabetes is associated with cardiovascular disease and microvascular pathologies in the retina, kidney and peripheral nerves [1], [2]. Most of these diabetic complications find their origin in damaging of the endothelium, a layer of cells lining the cardiovascular sytem [3], [4], [5]. The endothelium participates in numerous normal physiological functions including control of vasomotor tone, maintenance of blood fluidity, regulation of permeability, formation of new blood vessels and trafficking of cells. The endothelium also plays an important role in several human diseases. During inflammation, genes become activated within the endothelium to facilitate recruitment, attachment, and transmigration of inflammatory cells. In chronic inflammatory diseases, endothelial cell responses become impaired, leading to endothelial dysfunction (ED) [1], [6].

Erythritol (1,2,3,4-butanetetrol; ERT) is a natural C4 polyol that has a sweetness of 60–80% that of sucrose. More than 90% of ingested ERT is not metabolized by humans and excreted unchanged in urine, indicating ERT is efficiently absorbed not metabolized for energy and excreted by renal processes [7], [8]. It is a suitable bulk sweetener because it is not metabolized, does not influence blood glucose or insulin levels and does not cause caries [9], [10], consequently it is also safe for diabetics.

We have previously shown that ERT is an excellent hydroxyl radical scavenger *in vitro* and that it also delayed radical-induced hemolysis in red blood cells [11]. Supplementation with ERT reduced lipid peroxidation [8] and prevented loss of endothelium-dependent vasorelaxation in a diabetic rat model [11]. Given the importance of the endothelium in regulating vascular function and initiation and propagation of inflammatory responses to high glucose, herein, we, extend our previous studies with rats [11], by evaluating effects of ERT in an endothelial cell line exposed to normal and high glucose concentrations, using targeted and transcriptomic approaches.

## Materials and Methods

### Chemicals

Erythritol was kindly provided by Cargill (Vilvoorde, Belgium). F12K medium and fetal calf serum (FCS) were obtained from ATCC (Wesel, Germany). Penicillin/streptomycin, Hank's Balanced Salt Solution (HBSS) and trypsin were purchased from Gibco (Breda, The Netherlands). Glucose, N^G^-nitro-L-arginine methyl ester (L-NAME), 2-thiobarbituric acid (TBA), phosphoric acid, Ethylenediaminetetraacetic acid (EDTA), butylated hydroxytoluene (BHT), ethylene glycol tetraacetic acid (EGTA), nuclease P1, alkaline phosphatase, calcium ionophor A23187 and 4,5-diaminofluorescein diacetate (DAF-2) were obtained from Sigma Aldrich (Steinheim, Germany). 3-morpholino sidnonimine (SIN-1) was acquired from Alexis Biochemicals (San Diego, CA, USA). Endothelial cell growth supplement (ECGS) was obtained from BD Bioscience (Breda, The Netherlands). Heparin was purchased from Leo Pharmaceuticals (Amsterdam, The Netherlands). Ethanol, methanol and butanol were acquired from Biosolve (Valkenswaard, The Netherlands). [^3^H]-arginine was obtained from Perkin Elmer (Waltham, MA, USA).

### Cell Culture

Human umbilical vein endothelial (HUVEC) cell line CRL-1730 was obtained from ATCC. HUVECs were cultured in F12K medium with 10% non-heat inactivated FCS, 1% penicillin/streptomycin, 0.05 mg/ml ECGS and 0.1 mg/ml heparin. Cells were maintained in collagen coated T75 flasks (Greiner Bio-one, Alphen a/d Rijn, The Netherlands) at 37°C in a 5% CO2 atmosphere. For experiments, cells were seeded in 6 well plates/T75 flasks and grown until 80% confluency. Next, medium was removed and cells were washed with HBSS. New medium without supplements and erythritol (final concentration 5 mM), L-NAME (final concentration 0.1 mM or 0.5 mM) or vehicle solution (medium) was added to the cells. After 1 hour incubation glucose (final concentration 30 mM glucose) or vehicle (medium) was added to the cells. Subsequently, cells were incubated for 24 hours. The same protocol was used for incubation with SIN-1 (final concentration 0.5 mM).

### Cell viability

HUVEC cells were grown in 6 well plates until 80% confluence. After incubation medium was removed and the cells were washed HBSS and harvested with trypsin. All cell material including medium and HBSS was collected and centrifuged (5 minutes, 500×*g*) and used to determine viability of the cells using the trypan blue exclusion assay. The percentage of dead cells was calculated with the formula: (dead cells/(dead cells + viable cells) * 100%.

### Malondialdehyde measurement

Malondialdehyde (MDA) was measured with HPLC. Briefly, 100 µl of cell lysate or MDA standard were mixed with 1 ml of reagent, composed of 10 parts reagent A (12 mM TBA, 0.32 M phosphoric acid and 0.01 mM EDTA) and one part of reagent B (1.5 mg/ml BHT in ethanol). Samples and standards were heated for 1 hour at 99°C. After cooling, 500 µl of butanol was added and samples and standards were centrifuged for 5 minutes at maximum speed to extract the TBA-MDA product. Ten µl of the extract was injected on to an Alltima HP C18 column (Grace, Breda, The Netherlands) and eluted with 65% water and 35% methanol with 0.1% trifluoroacetic acid. Fluorescence was recorded at λ_ex_ 532 nm/λ_em_ 553 nm. MDA concentration was determined by calculating the peak height of the TBA-MDA product and results were corrected for protein content of the lysates.

### Protein carbonyl measurement

Cell lysates were monitored for their protein carbonyl contents using the protein carbonyl assay kit (Cayman Chemical, Ann Arbor, MI, USA). 2,4-Dinitrophenylhydrazine (DNPH) reacted with protein carbonyls in the lysate. The amount of protein-hydrozone produced was then quantified spectrophotometrically at an absorbance of 385 nm. The carbonyl content was corrected for protein content of the lysates.

### 8-Hydroxydeoxyguanosine measurement

DNA was extracted from HUVEC cells using the QIAamp DNA Mini Kit (Qiagen, Venlo, The Netherlands) according to the manufacturer's protocol and quantified spectrophotometrically. After extraction, 15 µg DNA was digested into deoxyribonucleosides by treatment with nuclease P1 (0.02 U/µl) and alkaline phosphatase (0.014 U/µl). To measure oxidative damage of DNA by 8-OHdG the Bioxytech 8-OHdG-EIA kit (Oxis Health products, Beverly Hills, CA, USA) was used. Digested samples were added to the microtiter plate precoated with 8-OHdG and the assay was performed according to the manufacturer's instructions.

### NOS3 activity

NOS3 activity was determined as described previously [12], [13], [14] using the NOS activity assay kit from Cayman. NOS3 activity was determined in cell pellets which were homogenized in ice-cold 25 mM Tris-HCl buffer containing 1 mM EDTA and 1 mM EGTA. Next, 22 µM [^3^H]-arginine (specific activity: 43 Ci/mmol) and 1 mM calcium chloride, 6 µM tetrahydrobiopterin, 2 µM flavin adenine mononucleotide and 1 mM of reduced nicotinamidedinucleotide phosphate as co-factors was added to to the homogenate. After 60 minutes incubation at room temperature, the reaction was stopped by adding a slightly acidic HEPES buffer containing a calcium ion chelator. [^3^H]-arginine was separated from [^3^H]-citrulline by DOWEX ion exchange resin. Scintilliation fluid was added and samples were counted for 5 minutes in a Wallac Liquid Scintillation counter. Background counts were determined by adding [^3^H]-arginine to the DOWEX resin and determining the remaining counts. Total counts were obtained by adding [^3^H]-arginine to the HEPES buffer and determining the counts. NOS3 activity was then determined by calculating the conversion percentage by % conversion = ((dpm reaction - dpm background)/dpm total)×100 after which the formed amount of [^3^H]-citrulline could be calculated. This value was then transformed into units of NOS3 activity per milligram protein. (1 unit = 1 micromole of citrulline per minute).

### Nitric oxide release

Quantification of nitric oxide (NO) released by the HUVEC was performed by using the DAF-2 fluorescence assay as described by Rathel et al [15]. HUVECs were grown in 6 well plates until 80% confluence. After incubation cells were washed twice with PBS + Ca^2+^. Subsequently, cells were incubated with PBS + Ca^2+^ containing 100 µM L-arginine for 10 minutes at 37°C. Afterwards, the calcium ionophor A23187 and DAF-2 were added into the buffer at final concentrations of respectively 1 µM and 0.1 µM. Next, cells were incubated in the dark for another 30 minutes at 37°C. Cell supernatants were then transferred into an opaque 96 well and fluorescence was measured on a spectrofluorometer (Spectra Max M2, Molecular Devices) with λ_ex_ set at 495 nm and λ_em_ at 515 nm. The NO release was corrected for protein content of the measured wells.

### Gene expression analysis

RNA was isolated from Qiazol suspended cells according to the manufacturer's protocol and quantified spectrophotometrically. Reverse transcription reaction was performed using 500 ng of RNA, which was reverse-transcribed into cDNA using iScript™ cDNA synthesis kit (Biorad, Veenendaal, The Netherlands). Next, real time PCR was performed with a BioRad MyiQ iCycler Single Color RT-PCR detection system using Sensimix™Plus SYBR and Fluorescein (Quantace-Bioline, Alphen a/d Rijn, The Netherlands), 5 µl diluted (10×) cDNA, and 0.3 µM primers in a total volume of 25 µl. PCR was conducted as follows: denaturation at 95°C for 10 minutes, followed by 40 cycles of 95°C for 15 seconds and 60°C for 45 seconds. After PCR a melt curve (60–95°C) was produced for product identification and purity. β-actin was included as internal control. Primer sequences for β-actin were: forward 5′-CCTGGCACCCAGCACAAT-3′ and reverse 5′-GCCGATCCACACGGAGTACT-3′ and for NOS3 forward 5′-GAGGGGAGCTGTTGTAGGG-3′ and reverse 5′-GTGGTAACCAGCACATTTGG-3′. Data were analysed using the MyIQ software system (BioRad) and were expressed as relative gene expression (fold change) using the 2^ΔΔCt^ method.

### Protein determination

Protein concentrations were determined spectrophotometrically using the DCprotein assay kit (BioRad) according to the manufacturer's protocol.

### Eicosanoid measurement

Eicosanoids (or oxylipins) derived from cyclooxygenase-, lipoxygenase- and cytochrome P450- enzymes, including those associated with hypertension and ED, were measured after published methods [16], [17] in cell pellets (nmol/g protein) and culture medium (nM). The 23 eicosanoids measured included 12,13-DiHOME, 9,10-DiHOME, 14,15-DiHETrE, 11,12-DiHETrE, 8,9-DiHETrE, 5,6-DiHETrE, 9(10)-EpOME, 12(13)-EpOME, 14(15)-EpETrE, 11(12)-EpETrE, 8(9)-EpETrE, 5(6)-EpETrE, TXB_2_, PGE_2_, PGD_2_, PGF2α, LTB_4_, 5-HETE, 8-HETE, 11-HETE, 12-HETE, and 15-HETE [for abbreviations, see Table S1 in [18]].

### RNA isolation and microarray experiments

Total RNA was isolated from Qiazol® suspended cells according to the manufacturer's protocol, followed by a clean-up, using a RNAeasy Mini Kit (Qiagen) with DNase treatment. RNA quantity and purity were determined spectrophotometrically using a Nanodrop. RNA quality was further assessed by automated gel electrophoresis on an Agilent 2100 Bioanalyzer (Agilent Technologies, Amstelveen, The Netherlands). All samples were found to be pure and free of RNA degradation. Sample preparation, hybridization, washing, staining and scanning of the Affymetrix Human Genome U133 Plus 2.0 GeneChip arrays (Affymetrix, Santa Clara, CA, USA) were conducted according to the manufacturer's manual. Quality controls were within accepted limits.

### Data processing and statistical analysis

Microarray data was processed using R and packages from the Bioconductor repository, including affy [19], [20], [21]. Probe sets and annotations were updated using the Entrez Gene based re-annotation by the BrainArray group [22]. The RMA algorithm was used to obtain background corrected, normalized, and log-transformed intensities for each probe set [23]. Genes that had low intensity signals (2log 100) on each array were removed before further processing. Determination of differentially expressed genes between relevant experimental groups was performed using the R limma package [24]. Regression models were built correcting for the day of the run and including an interaction term between ERT treatment status and glucose level.

### Data mining

Commercial and public domain database tools were used to annotate the changed transcripts. These included: the Gene Ontology (GO) database Transcript2GO; GeneSpring (Agilent Technologies, Inc., Santa Clara, CA, USA) for promoter analysis, transport factors and conservative natural language processing on Mesh terms and key words; ExPASy for reactions; DAVID for enzyme EC linking; Reactome for reactions amongst transcipts; and PhosphoSitePlus and GeneCards for annotations and transcript descriptions. The two main effects examined in pathway analysis were high glucose (30 mM, HG) vs normal glucose (7 mM, NG) and particularly high glucose in combination with with pre/coincubation with 5 mM erythritol (HGERT) vs HG. Normal glucose in combination with pre/coincubation with ERT vs NG was investigated minimally for pathway and network analysis. Pathway analysis was performed with PathVisio 2.0.7 [25] (www.pathvisio.org) using filtered microarray expression data and pathway collections from KEGG and WikiPathways (www.wikipathways.org). GeneSpring was also utilized to identify major pathways.

### Statistical analysis

For all analyses, p-values were calculated for the following comparisons: HGERT vs. HG (HGERT/HG); NGERT vs. NG (NGERT/NG); and HG vs. NG (HG/NG) (HG, high glucose; NG, normal glucose). For targeted analyses, there were 3 replications and data were evaluated by ANOVA models and student's t-tests for each of the above three comparisons. P-values<0.05 were considered statistically significant. P-values<0.1 were considered statistical trends, and are also described, since sample sizes were small (typically n = 3), and in some cases, assay variation was high.

## Results

### Erythritol attenuates glucose induced cell death

**Figure 1:**
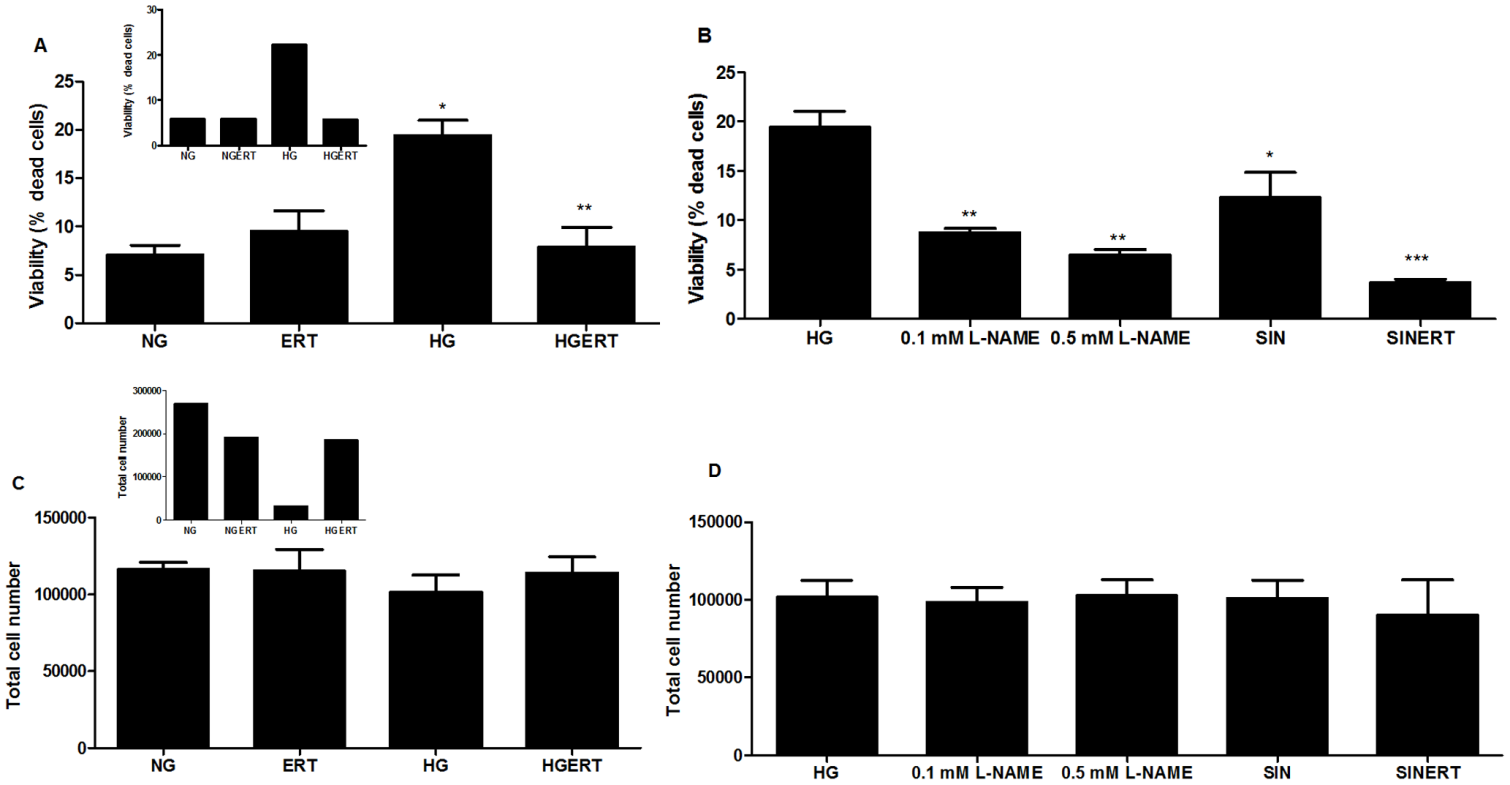
Erythritol attenuates cell death induced by diabetic stressors. Effect on viability of HUVECs incubated with normal glucose (NG, 7 mM) or high glucose (HG, 30 mM) in the presence or absence of erythritol (ERT, 5 mM) for 24 hours (A). Effect on viability of HUVECs incubated with HG in the presence N^G^-nitro-L-arginine methyl ester (L-NAME, 0.1 mM and 0.5 mM) and 3-morpholino sidnonimine (SIN, 0.5 mM) in the presence or absence of ERT (B) Effect of incubations on total cell number after 24 hours (C and D). Inset show data of 48 hour incubation with ERT, HG or HGERT (n = 1). Data are expressed as means ± standard error of at least three independent experiments. * = p<0.05 compared to NG; ** = p<0.05 compared to HG; *** = p<0.1 compared to SIN.

The effect of incubating HUVECs with HG, ERT or a combination of ERT and glucose (HGERT) was investigated by evaluating the cell viability using the trypan blue exclusion assay. When HUVECs were incubated with HG for 24 hours the percentage of death cells increased almost 4-fold (p = 0.0002) without affecting total cell number (Figure 1). Addition of ERT or the nitric oxide synthase (NOS) inhibitor L-NAME (0.1 mM or 0.5 mM) completely prevented this increase in the percentage of dead cells (p = 0.002 for ERT; p = 0.003 and p = 0.001 for L-NAME). Longer HG incubation (48 hours) resulted in a dramatically lower total cell number (inset figure 1C). Incubation for 24 hours with the peroxynitrite-generating compound SIN-1 also significantly increased cell death which was attenuated by addition of 5 mM ERT (p = 0.03 and p = 0.06). Moreover, under normal glucose conditions incubation with ERT did not result in an increased cell death compared to incubation without ERT.

### Effects on oxidative stress parameters

**Figure 2:**
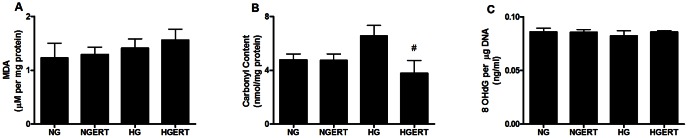
Effect on oxidative stress parameters. Effect of pre/co incubation with 5 mM erythritol (ERT) on HUVECS cultured in normal glucose (NG, 7 mM) or high glucose (HG, 30 mM) for 24 hours on malondialdehyde (A), carbonyl (B) and 8-OHdG (C) content. Data are expressed as means ± standard error of three independent experiments. # = p<0.1 compared to HG.

Because hyperglycemia is strongly associated with oxidative stress, we investigated three parameters of oxidative stress. Firstly, the protein carbonyl content of the HUVECs was measured. Protein carbonyls are products of the reaction between proteins and reactive oxygen species. Though not significant, a trend toward higher carbonyl content visible after incubation with HG compared to NG incubation for 24 hours (figure 2B). Addition of 5 mM ERT showed a trend toward a lower protein carbonyl content (p = 0.09). Next, the amount of malondialdehyde (MDA) in HUVECs was assessed. MDA is one of the end products of lipid peroxidation, a chain reaction in membrane lipids initiated by reactive oxygen species. Figure 2A indicates that incubation with 5 mM ERT, HG and HGERT does not increase the amount of MDA compared with HUVECs incubated with NG. Finally, the amount of oxidized nucleotide, in the form of 8-hydroxydeoxyguanosine (8-OHdG) was determined. Incubation with HG for 24 hours did not increase the amount of 8-OHdG (figure 2C). Furthermore, incubation with 5 mM ERT with either NG or HG did not have an effect on the amount of 8-OHdG in HUVECs.

### Effects on endothelial function

**Figure 3:**
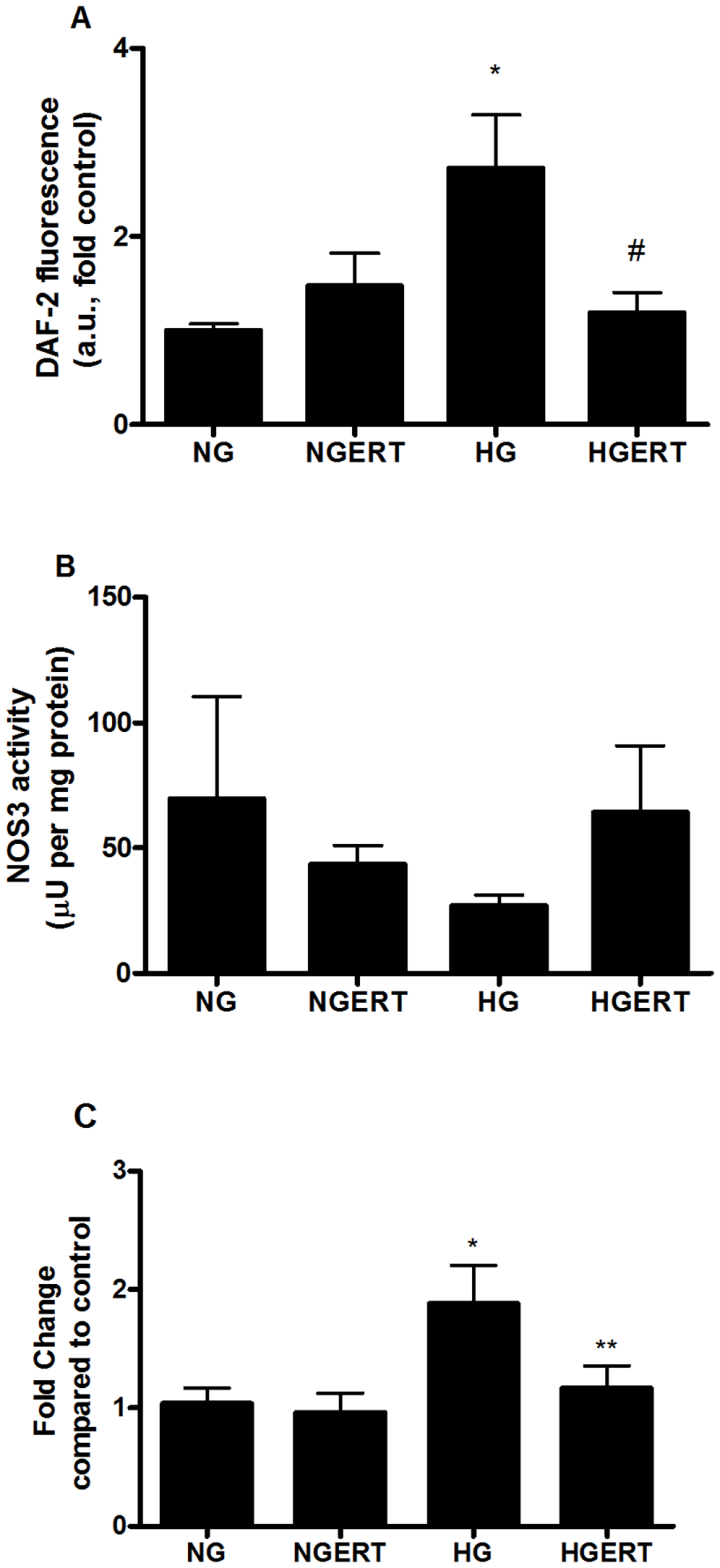
Effect on endothelial cell parameters. Effect of pre/co incubation with 5 mM erythritol (ERT) on HUVECS cultured in normal glucose (NG, 7 mM) or high glucose (HG, 30 mM) for 24 hours on NO release (A) NOS3 activity (B) NOS3 gene expression (C). Data are expressed as means ± standard error of at least three independent experiments. * = p<0.05 compared to NG; ** = p<0.05 compared to HG; # = p<0.1 compared to HG.

Production of the vasoactive gaseous radical nitric oxide (NO) by NOS is one of the most important functions of the endothelium. In the endothelium this is predominantly the NOS3 isoform [3], [26]. Therefore, we investigated the production of NO by HUVECs, which is shown in Figure 3A. When HUVECs were exposed to HG for 24 hours a 3-fold increase in NO release was observed (p = 0.04). Pre/coincubation with ERT showed a trend toward lower NO production (p = 0.06) compared to HG alone. Additionally, we looked at the effect of ERT on NOS3 activity in lysates from HUVECs exposed to HG (figure 3B). No difference between the conditions was observed. High variability (either biological or assay specific) may have prevented changes from being statistically different. Figure 3C shows an increase in gene expression of NOS3 after 24 hours under HG conditions (p = 0.03), which was attenuated by ERT (p = 0.1).

### Eicosanoid analysis

**Figure 4:**
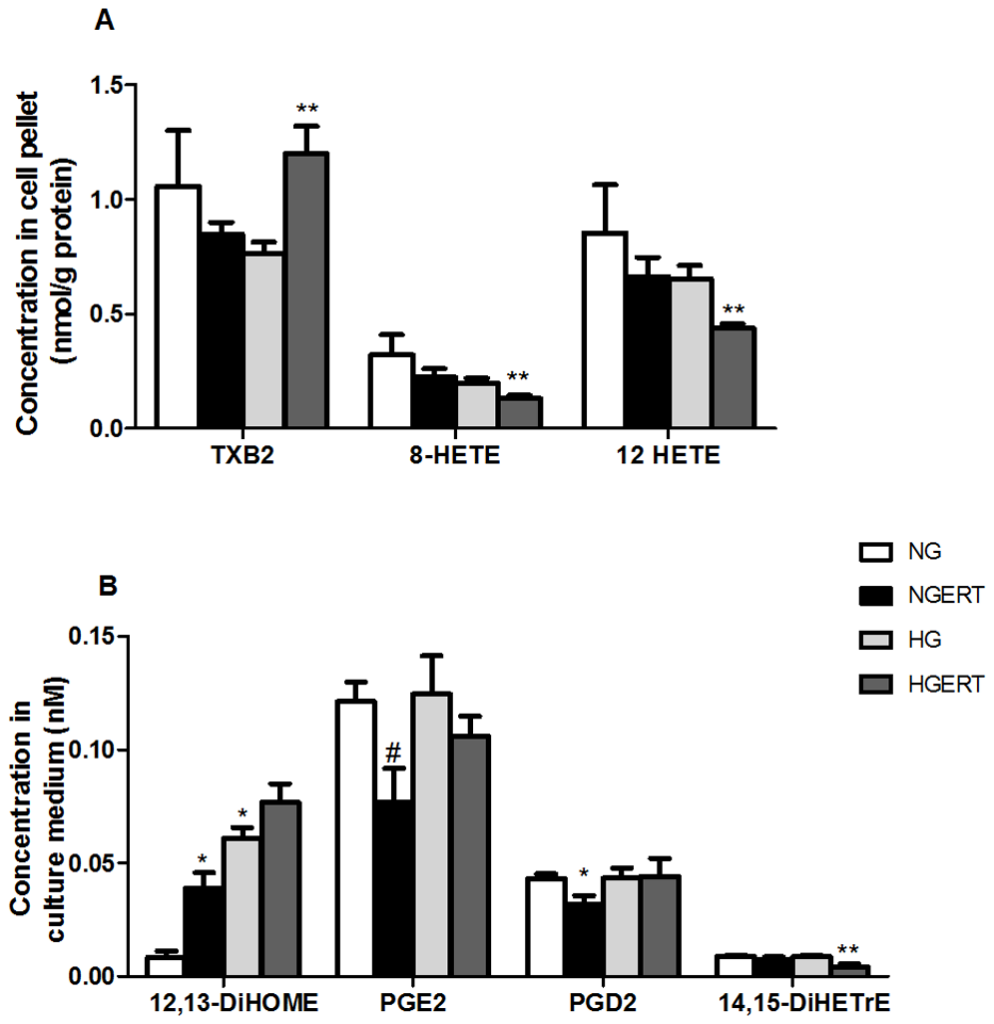
Effect on eicosanoid concentrations. Effect of pre/co incubation with 5 mM erythritol (ERT) on HUVECS cultured in normal glucose (NG, 7 mM) or high glucose (HG, 30 mM) for 24 hours on eicosanoid concentrations in cell pellets (A) and culture medium (B). Data are expressed as means ± standard error of three independent experiments. * = p<0.05 compared to NG; ** = p<0.05 compared to HG; # = p<0.1 compared to HG.

Eicosanoids formed from polyunsaturated fatty acids via classical cyclooxygenase and lipoxygenase pathways, as well as P450-derived epoxyeicosatreinoic acids (EETs) formed via soluble epoxide hydrolase (sEH) were measured in both cell pellets and culture medium (Figure 4 and table S1). Thromboxane B_2_ (TXB_2_) was increased in pellets of cells exposed to HGERT compared to HG alone in pellets (p = 0.03). Both 8-HETE (p = 0.05) and 12-HETE (p = 0.03) were decreased in pellets from cells exposed to HGERT compared to HG alone. In supernatants we only found a decrease of excretion of 14,15-dihydroxy-5Z,8Z,11Z-eicosatrienoate (14,15-DiHETrE) by cells exposed to HGERT compared to cells exposed to only HG (p = 0.04). Cells incubated with ERT excreted more 12,13-Dihydroxyoctadecenoic acid (12,13-DiHOME; p = 0.01) and showed a trend towards less prostaglandin E_2_ (PGE_2_; p = 0.06) and prostaglandin D_2_ (PGD_2_; p = 0.05) excretion compared to cells incubated without ERT.

### Transcriptomic analysis

**Figure 5:**
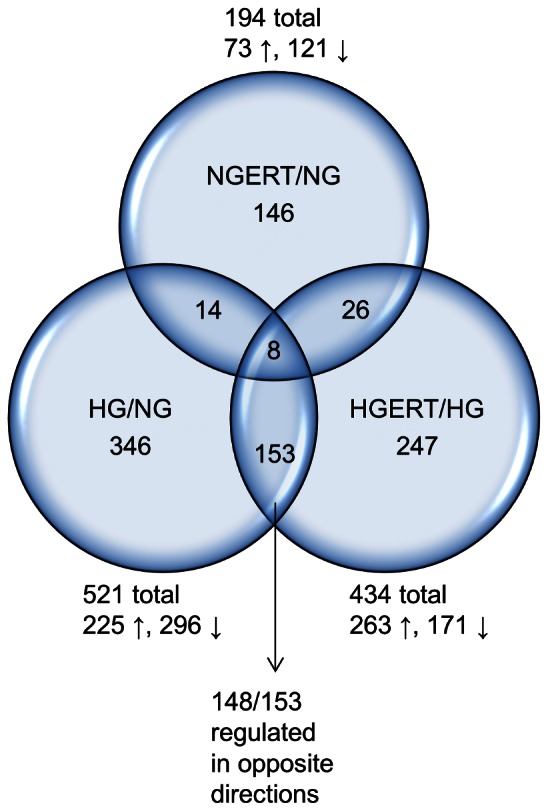
Venn diagram of changed transcripts. Venn diagram showing the overlap of differentially expressed transcripts after pre/co incubation with or without 5 mM erythritol (ERT) of HUVECs cultured in normal glucose (NG, 7 mM) or high glucose (HG, 30 mM) for 24 hours. Changed transcripts of the following comparisons are shown: HGERT vs HG (HGERT/HG); NGERT vs NG (NGERT/NG) and HG vs NG (HG/NG).

**Figure 6:**
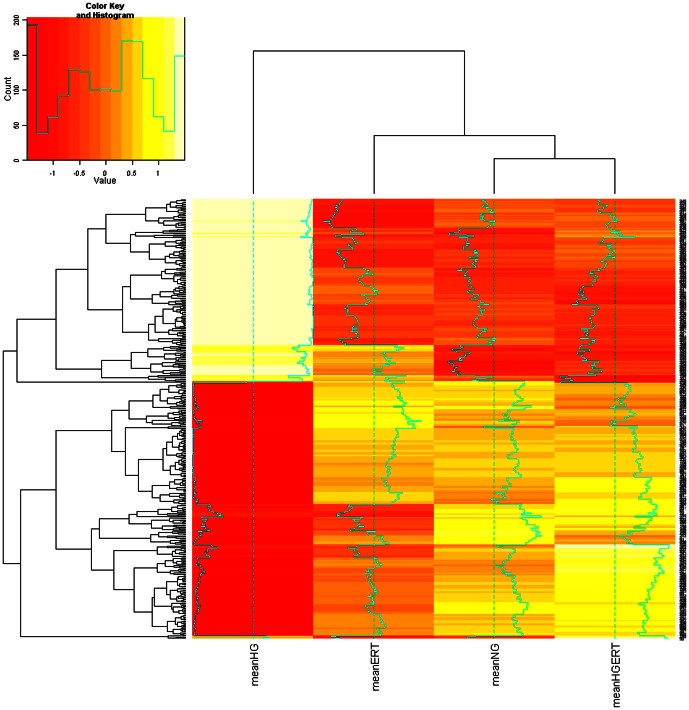
Heat map of transcriptomic analysis. Heat map reflecting the mean gene expression values in the four different treatment groups: From left to right: high glucose (HG, 30 mM), normal glucose and 5 mM erythritol (NGERT), normal glucose (NG), high glucose and 5 mM erythritol (HGERT). Cluster analysis shows that the expression profile in the HG group differs from the other three treatment group that form a separate cluster.

The numbers and overlap of transcripts changed in response to three comparisons are shown by Venn diagram (Figure 5). ERT induced small but significant fold changes in many transcripts. Maximum fold changes for down regulation were 0.94–0.97; and for up regulation were 1.04–1.13. There were 521 transcripts changed in response to HGERT vs. HG (HGERT/HG; p<0.05). Numbers of transcripts down- and up regulated was similar (296 down, 225 up). Comparing NGERT to NG (NGERT/NG), 194 transcripts changed. Only 6 transcripts changed in common for HGERT/HG and NGERT/NG, often with a different directionality, and did not change in response to HG/NG. Without ERT, HG alone (HG/NG) altered 434 transcripts. A striking observation was that under HG conditions, ERT reversed direction of change in 148 of the 153 transcripts changing in common with HGERT/HG and HG/NG, suggesting potential benefits of using ERT to ameliorate pathologies associated with hyperglycemia (figure 6). A subset of transcripts (368) were uniquely affected by HGERT/HG but not HG alone (HG/NG).

## Discussion

With this study we want to identify the mechanism(s) by which ERT exerts its endothelium-protective effect during diabetic stress, previously demonstrated in a diabetic rat model [11]. Hydroxyl radical scavenging by ERT alone cannot explain the powerful *in vivo* protective effects. Therefore the potential protective effects of ERT were investigated in different areas via targeted (e.g. cell viability, oxidative stress parameters, endothelial function parameters) and transcriptomic profiling in HUVECs. This cell line was chosen as a model because it has been used in a number of scientific studies into vascular inflammation, endothelial dysfunction and effects of hyperglycemia [27], [28], [29], [30], [31].

The induction of apoptotic endothelial cell death by HG has often been described [32], [33] and is highly implicated in the development of diabetic complications. We showed that exposure of HUVECs to HG increased the number of dead cells, which could be prevented by ERT. This higher number of death cells under HG conditions seems to be caused by an increase in NO, because addition of the NOS inhibitor L-NAME under HG conditions decreased the amount of death cells. The involvement of NO in glucose toxicity has been described previously [34], [35], [36]. Another indication of the involvement of NO in endothelial cell death was found when HUVECs were incubated with the peroxynitrite generator SIN-1. We showed that SIN-1 induced cell death, which was attenuated by ERT. Specifically for endothelial cells during diabetes, this is an important finding since peroxynitrite formation is likely to be increased during diabetes. Peroxynitrite is generated by the reaction of superoxide radicals with nitric oxide [37], the production of these precursors is known to be increased during diabetes [38], [39]. Peroxynitrite can induce lipid peroxidation and protein nitrosylation and thus plays a role in diabetes related tissue damage [40]. In a previous study, ERT was shown to have peroxynitrite scavenging activity in an *in vitro* system [41].

Subsequently, we looked at the ability of ERT to reduce oxidative damage caused by HG in HUVECs. Many studies have demonstrated that hyperglycemia triggers oxidative stress and generation of free radicals [1], [33], [42], [43]. These radicals cause damage to membranes, proteins and DNA resulting in cellular dysfunction and death. Radical scavenging by ERT reduces damage which may contribute to its endothelial protective effect. In HUVECs exposure to HG resulted in higher protein carbonyl levels while MDA and 8OHdG levels were not increased. This indicates that oxidative damage in HUVECs due to HG is concentrated in the cytosol. Since the majority of the proteins in the cell are located in the cytosol and therefore in the vicinity of the source of the high-glucose-induced oxygen radicals, it is likely that oxidative damage will probably be noted first as oxidized proteins as we observed with these results.

ERT did not affect NOS3 activity in HUVECs. Remarkably, the release of nitric oxide and the expression of the NOS3 gene were increased after incubation with high glucose only. This is in perfect agreement with the observation of Pandolfi and many others, who observed that HUVECs from human and animal origin, display increased NO production and NOS3 gene expression [39], [44]. How this relates to endothelial dysfunction, which is commonly regarded to be the result of impaired NO production, is currently unknown, although it has been suggested that the increased NO levels influence the transcription of genes that affect adenosine uptake by endothelial cells [39].

Eicosanoids are potent inflammatory mediators triggered by oxidative stress and/or hyperglycemia. Even small changes in amount of these bioactive molecules could be biologically important. Differences in concentration of TXB_2_, 8-HETE and 12-HETE were observed in cell pellets. Especially the decrease of 12-HETE in presence of ERT is of interest since it is a pro-inflammatory molecule produced from arachidonic acid via 12-lipoxygenase (12-LO) [45]. Oxidative stress and HG incubations of endothelial cells have been shown to increase 12-HETE and diabetic pigs with elevated blood glucose have increased 12-HETE [46]. In monocytes, HG increased 12-HETE and monocyte adhesion to endothelial cells via monocytic production of integrins [47]. In endothelial cells, 12-HETE induced integrin production in a PKC-dependent manner [48]. Exposure of endothelial cells to 12-HETE decreased production of vasodilatory PGI_2_ [49]. In culture medium we found differences in 14,15-DiHETrE which is produced from arachidonic acid via Cyp 2C and 2J to form EETs, which are in turn converted to DiHETrE via sEH. The decrease in 14,15-DiHETrE we found is consistent with HG suppression of sEH [50], resulting in increased EETs and EET-induced vasodilation. EETs were not observed to be increased in our system. Comparing ERT exposed cells to non-ERT exposed cells we also found some differences in the supernatants between molecules involved in mitochrondial dysfunction (12,13-DiHOME) and vasodilation and inflammation (PGE_2_ and PGD_2_) [51]. These findings indicate that various biologically important eicosanoids may mediate ERT effects under both NG and HG conditions in HUVEC cells.

To explore how ERT affected HUVECs on a transcriptional level we performed microarray analysis. We found several transcripts related to endothelial function to be altered when comparing HG to NG incubations including Bmp4, Vegfc and Ccl2 (table 1). Bmp4 is a member of the bone morphogenetic protein family, which is a part of the TGFβ superfamily of growth and differentiation factors. In endothelial cells, BMP4 produces a pro-inflammatory gene product inducing icam-1 and monocyte adhesion via NFκB signaling [52]. When overexpressed, BMP4 may contribute to endothelial dysfunction, promoting ROS production and apoptosis [53]. Vegfc is a PDGF/VEGF family member with roles in angiogenesis and endothelial cell growth. Ccl2 transcribes a chemotactic factor attracting monocytes and basophils. Other transcripts are involved in endothelial aggregation (pear1 [54]) and vasodilation (edn1). Also, HGERT and HG comparisons resulted in altered transcripts linked to endothelial function. These transcripts were involved in apoptosis (bmp6, highly expressed in HUVECs [55]), focal adhesion (jup, foxc1, krit1), differentiation and proliferation (notch1).

**Table 1:** Altered transcripts with a link to endothelial function.

Entrez gene name	Transcript	HGERT/HG	HG/NG
Chemokine (C-C motif) ligand 2	ccl2		1.04
Endothelin 1	edn1		0.99
Endoglin	eng	1.01	0.98
Forkhead box C1	foxc1	1.01	
Growth factor receptor-bound protein 10	grb10		0.99
KRIT1, ankyrin repeat containing	krit1	0.99	
Notch 1	notch1	1.02	
Platelet endothelial aggregation receptor 1	pear1		0.99
Ras homolog gene family, member J	rhoj	1.02	
Tumor necrosis factor, alpha-induced protein 1 (endothelial)	tnfaip1		0.99
Vascular endothelial growth factor C	vegfc		1.04
Bone morphogenetic protein 4	bmp4		0.99
Bone morphogenetic protein 6	bmp6	1.01	

Transcripts related to apoptosis are shown in table 2. Under HG, ERT signalled via numerous pro- and anti-apoptotic pathways. As ERT protects endothelial cells from cell death under HG conditions (figure 1A), it seems that ERT has anti-apoptotic effects and that post translational modifications of transcribed proteins and dimerization events may explain why pro-apoptotic transcriptomic changes seem to have occurred (Table 2).

**Table 2:** Altered transcripts with a link to apoptosis.

Anti-apoptotic				Pro-apoptotic			
Pathway	Transcript	HGERT/HG	HG/NG	Pathway	Transcript	HGERT/HG	HG/NG
AKT/Bad	pik3r1	1.03		Caspase	hip1	1.05	
AKT/FRAP1	ddit4l	0.97		Cell cycle	maged1	1.01	
BLK	elf2	0.99	1.01	Cell cycle/CDK	ccni	1.01	
Caspase	hspe1	0.99		Cell proliferation	pdcd7	1.02	0.99
Caspase	ifi6	1.13		Cell proliferation	ubn1	1.02	
Cell proliferation	furin	1.02		DNA repair	rrm2b	0.98	
DNA repair	actr5	1.01		FOX	foxn3	1.01	0.99
FOX	foxc1	1.01		FOX	foxp1	1.01	
Impedes cyt c release	gsn	1.03		HER-2/NEU	casc4	0.99	1.01
JNK/SAPK	mbip	0.98		JNK/SAPK	map4k3	0.98	
p38 MAP kinase	stk39	0.98		JNK/SAPK	sos1	1.06	0.94
P53/XIAP inhibition	notch1	1.02		MYC family	mxd4	1.02	0.98
RAS	rsu1	0.99		P53/CDK	ccnk	1.01	
RAS	rhob	1.02	0.97	P53	rybp	1.01	0.99
RAS	rhoj	1.02		P53	tp53bp2	1.01	
RAS	rab3b	1.07		P53	tbrg1	1.02	0.98
TGFβ	acvr2a	0.99		P53	tp53i11	1.02	0.98
TGFβ	bmp6	1.01		RAS	rassf2	1.01	1.03
	eng	1.01	0.98	WNT	hbp1	0.99	
	atxn3	0.99			dap	1.01	
	pdcd6	0.99			serinc3	1.01	0.99
	socs3	1.01			sox4	1.01	0.99
	txndc5	1.03			lyn	1.02	0.99

Over-represented canonical pathways included (table 3): tricarboxylic acid cycle (TCA) cycle, TGF beta signaling pathway, glutathione metabolism and glucuronidation. Noncanonical networks included PIK3R1, NFκB, HNF, XBP1, SOS, and RELA. These canonical and non-canonical pathways are linked to diabetes onset, insulin signaling and production of adhesion molecules/nitric oxide.

**Table 3:** Top 10 pathways regulated by exposure of HUVECs to high glucose (HG effect) or to erythritol during exposure to high glucose (HGERT effect).

Pathways regulated by exposure to high glucose	Z Score
TGF Beta Signaling Pathway	3.95
Benzo(a)pyrene metabolism	3.35
Pentose and glucuronate interconversions	3.26
Glycosylphosphatidylinositol(GPI)-anchor biosynthe	3.07
Diurnally regulated genes with circadian orthologs	3.05
Prostate cancer	2.92
Sphingolipid metabolism	2.75
Pathways in cancer	2.58
Antigen processing and presentation	2.48
Caffeine metabolism	2.44

Pathways regulated by erythritol during exposure to high glucose	Z Score
Chronic myeloid leukemia	4.03
Citrate cycle (TCA cycle)	3.88
Delta-Notch Signaling Pathway	3.78
Prostate cancer	3.24
Androgen Receptor Signaling Pathway	3.09
TGF-beta Receptor Signaling Pathway	2.86
Glutathione metabolism	2.77
Glucuronidation	2.61
B cell receptor signaling pathway	2.48
G13 Signaling Pathway	2.42

Of particular interest are changes in the TCA cycle and electron transport chain, changes of transcripts are shown in table 4. Pyruvate dehydrogenase (PDH) complex transfers the acetyl group of pyruvate to coenzyme A prior to the citric acid cycle. A very slight up regulation with HG/NG for pyruvate dehydrogenase (lipoamide) beta (pdhb), encoding the E1 beta subunit responsible for pyruvate dehydrogenase activity was observed. Branched-chain alpha-keto acid dehydrogenase complex (BCKD), analogous to the PDH complex, is an inner-mitochondrial enzyme complex responsible for the degradation of branched-chain amino acids (e.g. isoleucine, leucine, and valine). It converts α-keto acids to acyl-CoA + CO_2_ and requires thiamine pyrophosphate (TPP), FAD, NAD^+^, lipoate and coenzyme A as cofactors. BCKD complex contains 24 core transacylase (E2) subunit and associated decarboxylase (E1), dehydrogenase (E3) and regulatory subunits. The lipoamide acyltransferase (or transacylase) E2 subunit component of BCKD is encoded by dihydrolipoamide branched chain transacylase E2 (dbt). DBT was slightly up regulated with HGERT/HG. Succinate CoA synthetase converts succinyl CoA and ADP or GDP to succinate and ATP or GTP. Succinate-CoA ligase, ADP-forming, beta subunit (sucla2) was down regulated with HGERT/HG, and up regulated with HG/NG. Transcripts coding for other subunits such as suclg1 (αsubunit) and suclg2 (β subunit) were not affected by treatments. In the next reaction in the citric acid cycle, succinate dehydrogenase converts succinate to fumarate in an oxidation step. Succinate dehydrogenase is unique amongst citric acid enzymes, in that it is a nonheme iron protein located in the inner mitochondrial membrane, directly linked to electron transport. Two electrons from FADH_2_ are transferred to FeS clusters on the enzyme which are in turn transferred to ubiquinone (coenzyme Q) and then molecular oxygen. Fumurase then converts fumurate to malate, which is in turn oxidized to oxaloacetate by malate dehydrogenase, using the reduction of NAD^+^ to NADH. Ubiquinone binds in a gap between subunits B, C, and D. Succinate dehydrogenase (sdh) consists of 2 hydrophilic subunits (A, B) and 2 hydrophobic membrane anchor subunits (C, D) with phospholipid binding sites for cardiolipin (CL) and phosphatidylethanolamine (PE). Transcripts coding for the hydrophilic domains (sdha, sdhb) were not affected. Succinate dehydrogenase complex, subunit C and D (sdhc and sdhd) were down regulated with HGERT/HG, and up regulated with HG/NG.

**Table 4:** Transcripts changed in citric acid cycle and electron transport system.

Complex	Transcript	HGERT/HG	HG/NG
Pyruvate dehydrogenase	pdhb		1.01
Succinate CoA synthetase	sucla2	0.99	1.01
Succinate dehydrogenase	sdhc	0.96	1.02
	sdhd	0.98	1.01
Complex I NADH dehydrogenase	ndufa4	0.99	1.02
	ndufa12	0.99	
Complex II Succinate dehydrogenase	sdhc	0.96	1.02
	sdhd	0.98	1.01
Complex III Cytochrome bc1	qcr7 (uqcrb)	0.99	1.01
	qcr9 (ucrc, uqcr10)	0.99	
Complex IV cytochrome c oxidase	cox5b	0.98	
	cox7a2	0.99	
	cox8a	0.99	
	cox16	0.99	
	cmc1		1.01
Complex V ATP synthase	atp5e	0.99	1.01
	atp5o	0.99	
	atp5l		1.01

Electron transport occurs in the inner mitochondrial membrane via enzymatic reactions utilizing electron donors and acceptors. It is responsible for generation of ATP from products of the TCA cycle, fatty acid oxidation and amino acid oxidation. This pathway is tied to oxidative stress (and hyperglycemia via excess glucose equivalents entering the mitochondrial machinery) as a small percentage of electrons ‘leak out’ resulting in superoxide formation. Numerous transcripts involved in electron transport (11) had slight down regulation with HGERT/HG, five of these were oppositely regulated with HG/NG (Table 2). Transcripts were changed in all 5 electron transport chain complexes. In complex I, NADH dehydrogenase, the subunits ubiquinone 1 alpha, subcomplexes 4 (ndufa4) and 12 (ndufa12) were down regulated with HGERT/HG. Ndufa4 was up regulated with HG/NG. Overactivity of the mitochondrial respiratory chains occurs during hyperglycemia [56], increasing transcription of complex II. This in turn increases electron leaking and production of superoxide radicals. Subunits C and D of complex II were down regulated with HGERT/HG, and up regulated with HG/NG (see also citric acid cycle). This countering of up regulation by ERT under HG conditions probably reduces superoxide production. The ability of ERT to prevent HG-induced increases in SDH suggests ERT may protect the mitochondria from oxidative damage via this mechanism. The reduction of coenzyme Q in complex III (cytochrome bc_1_ complex) can also contribute to oxidant production as highly reactive ubisemiquinone free radicals are formed as intermediaries in the Q cycle, leading to electron leakage and superoxide radicals [57]. In complex III, Ubiquinol-cytochrome c reductase binding protein (uqcrb = qcr7; orthology to subunit 7) and ubiquinol-cytochrome c reductase, complex III subunit X (ucrc = uqcr10 = qcr9) were down regulated with HGERT/HG. uqcrb was up regulated with HG/NG. In complex IV, cytochrome c oxidase (COX), subunits VB- (cox5b), VIIa- (cox7a2), VIIIA- (cox8a), and 16 (cox16) were down regulated with HGERT/HG (and not affected with HG/NG). COX assembly mitochondrial protein homolog (*S. cerevisiae*) (cmc1) is required for mitochondrial COX assembly and respiration. It binds copper, and may be involved in copper trafficking and distribution to COX and superoxide dismutase 1 (SOD1) [58]. Cmc1 showed slight up regulation with HG/NG (not changed with HGERT/HG). In complex V, ATP synthase, H^+^ transporting, mitochondrial F_1_ complex, subunits- epsilon (atp5e) and 0 (atp5o) convert ADP to ATP, pumping protons across the proton-motive force. F_1_ complexes (and their 5 subunits) contain extra-membranous catalytic activity. F_0_ complexes contain the membrane-spanning component comprising the proton channel, and contain 9 subunits. Atp5e was down regulated with HGERT/HG, and up regulated with HG/NG; atp5o was very slightly down regulated with HGERT/HG. ATP synthase, H^+^ transporting, mitochondrial F_o_ complex, subunit G (atp5l) was slightly up regulated with HG/NG only.

Although there were considerable changes to electron transport transcripts, there was limited evidence from transcriptomic and targeted analyses that ERT acts like “classical” antioxidant in decreasing levels of oxidants via effects on glutathione peroxidases (gpx), peroxiredoxins (prdx), superoxide dismutases (sod), superoxides (alox, cyb, duox, ncf, nos), ROS metabolism and oxidative stress responsive genes. Based on transcript annotations, some transcripts are associated with ROS, including krit1, bmp4 and sh3pxd2b (increased), the latter with a role in NOX-dependent ROS production. Also, transcriptomic changes related to the citric acid cycle and electron transport chain suggest ERT may reduce mitochondrial superoxide production through a novel mechanism.

This study shows that erythritol has a large number of minor, often not reaching significance, beneficial effects in endothelial cells during exposure to high glucose. It is difficult to pin point a specific effect by which erythritol protects the cells during diabetic stress, and thus to explain why erythritol was capable of preventing the onset of endothelial dysfunction in the diabetic rat. However, it is more than likely, that the combination of all the effects displayed by erythritol is ultimately responsible for its extraordinary protective effect *in vivo*.

In conclusion, our present data point at a therapeutically important protective effect of ERT in endothelial cells. Overall, this study demonstrates that ERT by itself (i.e. under non-diabetic conditions) has minimal effects on HUVECs. Viability, oxidative damage, endothelial function parameters and the transcriptome do not show changes after incubation with ERT. However, when cells are exposed to HG following preincubation with ERT, a number of deleterious effects caused by HG are reversed. The observation that ERT does not affect single endpoints but has multi-targeted effects is not unusual for a natural compound. We have previously observed the same mode of action in other studies [59]. Therefore, it is expected that in non-diabetic subjects ERT will not affect the endothelium which is a desirable property, while in diabetic subjects where the endothelium is under diabetic stress, ERT could shift a variety of damage and dysfunction parameters to a safer side. ERT can therefore be regarded as a compound that has definite endothelium protective effects during hyperglycemia.

There is still a considerable need for safe agents that can reduce the risk of developing diabetic complications. These diabetic complications in general are the consequence of endothelium dysfunction. ERT can therefore be of great importance to a rapidly growing population of people with diabetes to reduce their risk of developing diabetic complications.

Because diabetes is a chronic disease, supplementation with antioxidants to prevent the onset and development of diabetic complications will be chronic as well. Compounds with strong and explicit biological activities are probably not indicated in long term protection during diabetes. It is therefore important to choose a compound that has mild protective effects in small vessel and arteries because the endothelial cells are an important target of hyperglycemic damage. This study shows that ERT exerts many such beneficial effects on endothelial cells during exposure to diabetic stressors.

## Supporting Information

Table S1 **Effect of pre/co incubation with 5 mM erythritol (ERT) on HUVECS cultured in normal glucose (NG, 7 mM) or high glucose (HG, 30 mM) on eicosanoids concentrations in cell pellets and culture medium.** Data are expressed as means ± standard error of three independent experiments. *p<0.05 compared to NG; **p<0.05 compared to HG.
(DOCX)
