# Supplementary material for: Multi-Targeted Mechanisms Underlying the Endothelial Protective Effects of the Diabetic-Safe Sweetener Erythritol
Source: PLoS One. 2013 Jun 5;8(6):e65741. doi: 10.1371/journal.pone.0065741 (PMC3673924; doi:10.1371/journal.pone.0065741)
Supplement: Table S1 — Effect of pre/co incubation with 5 mM erythritol (ERT) on HUVECS cultured in normal glucose (NG, 7 mM) or high glucose (HG, 30 mM) on eicosanoids concentrations in cell pellets and culture medium. Data are expressed as means ± standard error of three independent experiments. *p<0.05 compared to NG; **p<0.05 compared to HG. (DOCX) [file pone.0065741.s001.docx]

Supporting Table 1: Effect of pre/co incubation with 5 mM erythritol (ERT) on HUVECS cultured in normal glucose (NG, 7 mM) or high glucose (HG, 30 mM) on eicosanoids concentrations in cell pellets and culture medium. Data are expressed as means ± standard error of three independent experiments. * = p<0.05 compared to NG; **=p<0.05 compared to HG

|  | **Concentration in cell pellet**  **(nmol/g protein)** | | | | **Concentration in supernatant  (nM)** | | | |
| --- | --- | --- | --- | --- | --- | --- | --- | --- |
|  | **NG** | **NGERT** | **HG** | **HGERT** | **NG** | **NGERT** | **HG** | **HGERT** |
| 12,13-DiHOME | N.D. | N.D. | N.D. | N.D. | 0.01±0.00 | 0.04±0.01* | 0.06±0.01* | 0.08±0.01 |
| 9,10-DiHOME | N.D. | N.D. | N.D. | N.D. | N.D. | 0.01±0.00 | 0.01±0.01 | 0.01±0.01 |
| 14,15-DiHETrE | 0.01±0.01 | N.D. | N.D. | N.D. | 0.01±0.00 | 0.01±0.00 | 0.01±0.00 | 0.00±0.00** |
| 9(10)-EpOME | 0.05±0.01 | 0.05±0.03 | 0.02±0.01 | 0.03±0.02 | 0.02±0.00 | 0.02±0.01 | 0.02±0.02 | 0.02±0.01 |
| 12(13)-EpOME | 0.03±0.02 | 0.05±0.05 | 0.02±0.01 | 0.02±0.00 | 0.03±0.01 | 0.02±0.01 | 0.03±0.01 | 0.02±0.00 |
| 14(15)-EpETrE | 0.02±0.02 | 0.04±0.02 | 0.01±0.01 | 0.02±0.02 | 0.01±0.00 | 0.01±0.00 | 0.01±0.00 | 0.01±0.00 |
| 11(12)-EpETrE | 0.06±0.02 | 0.06±0.04 | 0.04±0.03 | 0.06±0.05 | 0.03±0.00 | 0.02±0.00 | 0.02±0.01 | 0.02±0.00 |
| 8(9)-EpETrE | 1.05±0.29 | 0.88±0.06 | 0.84±0.11 | 0.73±0.16 | 0.07±0.02 | 0.07±0.01 | 0.10±0.05 | 0.10±0.02 |
| 5(6)-EpETrE | 0.12±0.07 | 0.05±0.01 | 0.06±0.04 | 0.04±0.02 | 0.03±0.01 | 0.03±0.00 | 0.03±0.01 | 0.03±0.00 |
| TXB2 | 1.06±0.43 | 0.85±0.09 | 0.77±0.09 | 1.20±0.20** | 0.07±0.01 | 0.05±0.01 | 0.06±0.00 | 0.06±0.01 |
| PGE2 | 0.95±0.24 | 0.69±0.14 | 0.66±0.13 | 0.75±0.05 | 0.12±0.02 | 0.08±0.03* | 0.12±0.03 | 0.11±0.02 |
| PGD2 | 0.48±0.06 | 0.46±0.15 | 0.43±0.10 | 0.46±0.08 | 0.04±0.00 | 0.03±0.01* | 0.04±0.01 | 0.04±0.01 |
| PGF2a | 1.32±0.54 | 0.90±0.05 | 1.20±0.79 | 1.03±0.11 | 0.31±0.05 | 0.24±0.03 | 0.31±0.07 | 0.23±0.04 |
| 5-HETE | 2.22±0.99 | 1.60±0.61 | 1.33±0.12 | 1.18±0.09 | 0.02±0.00 | 0.02±0.00 | 0.02±0.01 | 0.02±0.00 |
| 8-HETE | 0.32±0.16 | 0.22±0.06 | 0.20±0.04 | 0.13±0.02** | N.D. | N.D. | N.D. | N.D. |
| 11-HETE | 1.74±0.34 | 1.40±0.10 | 1.36±0.33 | 1.64±0.29 | N.D. | N.D. | N.D. | N.D. |
| 12-HETE | 0.85±0.36 | 0.66±0.15 | 0.65±0.10 | 0.44±0.03** | 0.06±0.01 | 0.04±0.01 | 0.07±0.03 | 0.08±0.06 |
| 15-HETE | 3.25±1.14 | 2.32±0.36 | 2.36±0.36 | 2.08±0.16 | 0.05±0.02 | 0.05±0.01 | 0.08±0.04 | 0.06±0.02 |
| sum of EpOME | 0.07±0.03 | 0.10±0.08 | 0.05±0.02 | 0.05±0.02 | 0.04±0.01 | 0.04±0.01 | 0.06±0.02 | 0.04±0.01 |
| Sum of  EpETrE with 5,6 | 1.25±0.30 | 1.02±0.08 | 0.96±0.11 | 0.84±0.18 | 0.13±0.02 | 0.13±0.00 | 0.17±0.06 | 0.16±0.02 |
| sum of EpETrE without 5,6 | 1.13±0.30 | 0.98±0.08 | 0.90±0.08 | 0.80±0.20 | 0.10±0.02 | 0.10±0.00 | 0.14±0.06 | 0.13±0.02 |
| sum of HETE | 10.12±3.24 | 7.61±1.19 | 7.27±1.12 | 7.10±0.76 | 0.12±0.04 | 0.11±0.02 | 0.17±0.08 | 0.16±0.09 |
